# Clinical outcomes of a hydrophobic trifocal diffractive intraocular lens: a literature review

**DOI:** 10.3389/fmed.2025.1533161

**Published:** 2025-01-22

**Authors:** Deborah Ristvedt, Camille Bosc, Vance Thompson

**Affiliations:** ^1^Vance Thompson Vision, Alexandria, MN, United States; ^2^Departement de Recherche Clinique, Institut Ophtalmologique de l'Ouest (IOO) Jules Verne, Nantes, France; ^3^Vance Thompson Vision, Sioux Falls, SD, United States; ^4^Sanford School of Medicine, University of South Dakota, Sioux Falls, SD, United States

**Keywords:** trifocal, intraocular lens, hydrophobic, diffractive, defocus curve

## Abstract

The aim of this review is to summarize the clinical outcomes reported in patients following the implantation of the hydrophobic diffractive trifocal FineVision HP intraocular lens (IOL). A literature search in PubMed (U.S. National Library of Medicine) was performed to identify publications, both prospective and retrospective, which have reported the clinical outcomes of patients who were implanted with this IOL model after cataract or refractive lens exchange surgeries. A total of 18 clinical studies published between 2019 and 2024 were included in this review. The available data reported in the different clinical publications was analyzed in detail, focusing mainly on refraction, visual acuity, the defocus curve, optical quality, contrast sensitivity, and patient-reported outcome questionnaires. In addition, the adverse events and other measurements reported in some studies were also analyzed. Our review encompassed a total of 1,028 eyes analyzed at different follow-up periods up to a maximum of 24 months. The analysis carried out in this review leads us to conclude that the FineVision HP IOL provides good refractive outcomes and excellent visual performance when implanted.

## 1 Introduction

Pseudophakic intraocular lenses (IOLs) for correcting presbyopia after cataract or refractive lens exchange (RLE) surgery have been developed in recent decades to provide patients with good visual acuity at different distances with less dependence on glasses. Various designs and models that provide more than one focal point have been proposed including bifocal, trifocal, and extended depth-of-focus (EDOF) IOLs. In general, these solutions have shown to be safe and predictable and afford good visual acuity, the results being compiled in various review publications (1–5). A Bayesian network meta-analysis compared the outcomes of the different presbyopia-correcting IOLs frequently used in clinical practice (1). That review included 27 studies comprising 2,605 patients and concluded that for patients considering a multifocal IOL due to presbyopia, the bilateral implantation of a trifocal IOL might be the optimal option, with limited compromise to distance visual acuity. A specific comparison between EDOF versus trifocal IOLs based on 22 studies recruiting 2,200 eyes showed that trifocal IOLs improve uncorrected near visual acuity compared to EDOF IOLs (2). A recent Cochrane meta-analysis comparing the two solutions also concluded that people receiving trifocal IOLs may achieve better near vision and may be less dependent on spectacles at this distance than those implanted with EDOF IOLs (5). The efficacy and safety of various presbyopia-correcting IOLs post-cataract surgery, including bifocal, trifocal, EDOF, and enhanced monofocal IOLs, have been evaluated in 28 randomized controlled trials comprising 2,465 subjects, and it has been concluded that for cataract patients who want to treat presbyopia, trifocal IOLs demonstrate better visual acuity and spectacle independence at near distances. Correcting astigmatism by adding a toric design to these lenses has been proved to be effective, allowing complete visual restoration over a wide range of distances (3). Trifocal IOLs may therefore be considered the best presbyopia-correcting IOL solution after cataract surgery.

**Figure 1:**
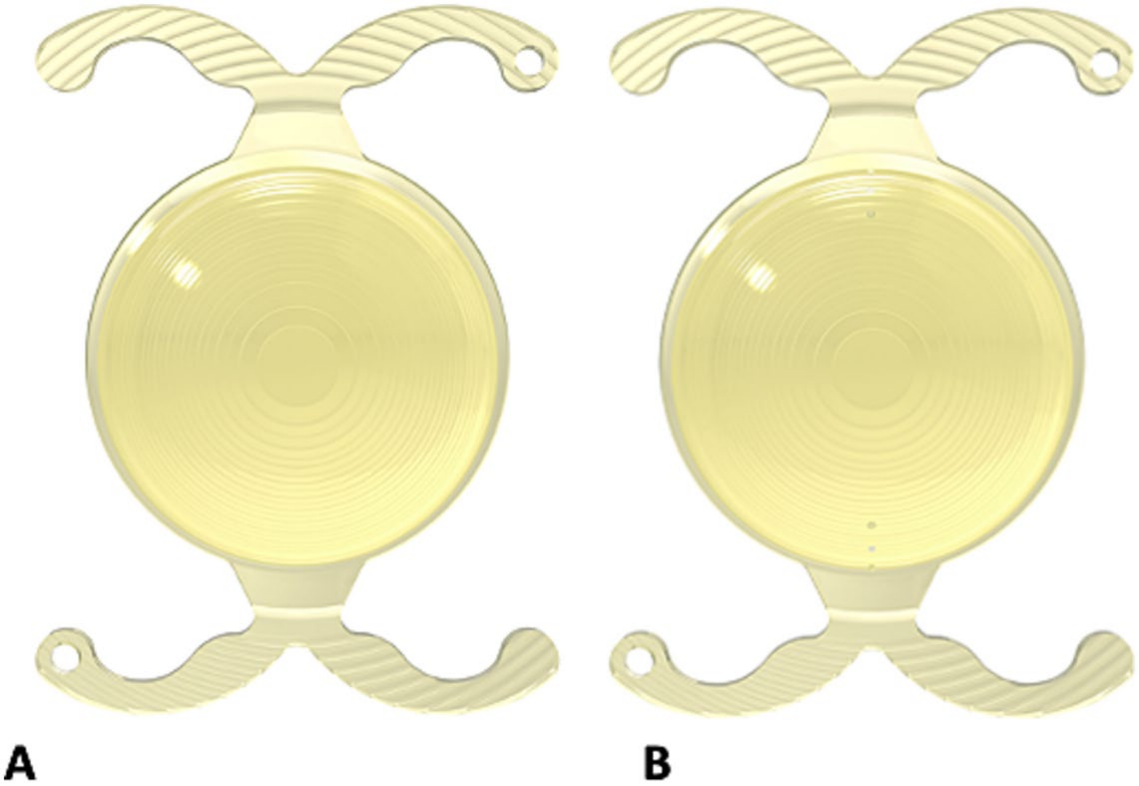
Images of the FineVision HP (POD F GF) intraocular lens [**(A)** non-toric model, and **(B)** toric model; courtesy of BVI Inc., Waltham, United States].

One of the most widely used trifocal lenses around the world is the FineVision IOL (BVI Inc., Waltham, United States). The most recent model of this lens appeared on the market in 2019: the FineVision HP (POD F GF, Figure 1); it was created using glistening-free hydrophobic acrylic material GF (6) and it uses the same optical diffractive trifocal design as in the hydrophilic FineVision model (POD F IOL). This lens has a double C-loop haptic design and is diffractive to create 2 additions: +3.50 D and + 1.75 D. The FineVision HP IOL has been used worldwide and several clinical studies carried out by surgeons in a number of countries have published their outcomes. These publications have analyzed several samples, follow-up periods and clinical metrics in order to demonstrate the safety and efficacy of this lens after cataract or RLE surgeries. To the best of our knowledge, no review of these studies has been carried out to date. Consequently, the main purpose of this report is to review the clinical outcomes that the trifocal FineVision HP IOL has yielded in the context of published studies in international peer-review journals.

## 2 Methods

We searched the PubMed database (U.S. National Library of Medicine) for publications of clinical studies that included the FineVision HP IOL whether implanted monocularly or binocularly. Only retrospective or prospective clinical studies published in peer-reviewed journals written in English were considered for this analysis. The date of the last electronic search was November 20, 2024. The search included a combination of any of the following keywords: ‘IOL’, ‘FineVision’, ‘POD F GF’, ‘trifocal’, ‘diffractive’ and ‘hydrophobic’. Moreover, for each selected article obtained from this search, all of its references were also checked to ensure that any clinical publication including this IOL would not be missed.

After this search, 18 articles were identified and subsequently analyzed in detail. The first paper reporting clinical outcomes of this IOL was published in 2019 and the last one in 2024. Specifically, 2 were published in 2019, 2 in 2020, 5 in 2022, 3 in 2023 and 6 in 2024. The main characteristics of each publication were extracted. The following information was considered: the name of the authors, publication year, country, article title, journal, number of eyes and patients recruited, type of study (prospective or retrospective), type of surgery (cataract or RLE), follow-up time (maximum), patient ages, IOL power (sphere and cylinder), axial length, corrected and uncorrected visual acuity at different distances (UDVA: uncorrected distance visual acuity; UIVA: uncorrected distance intermediate visual acuity; UNVA: uncorrected distance near visual acuity; CDVA: corrected distance visual acuity; DCIVA: distance-corrected intermediate visual acuity; and DCNVA: distance-corrected near visual acuity), refraction (sphere, cylinder, and spherical equivalent [SE]), the monocular and binocular defocus curve, optical quality (wavefront aberrations), photopic and mesopic contrast sensitivity, and patient-reported outcome questionnaires (PROQ). In addition, any adverse events dysphotopsia, glare, and halos, were also considered and analyzed. Whenever available, the mean, standard deviation, and ranges were included for all the parameters analyzed. In the case of reporting data at different follow-ups the outcomes assessed in this article were those for the longest postoperative visit.

## 3 Results

As previously indicated, the search, following the criteria indicated, resulted in a total of 18 articles published between 2019 and 2024 (7–24). These articles were thoroughly analyzed and Table 1 was created to show the main descriptive characteristics (mean, standard deviation, and range, when available): authors, year of publication, country, number of eyes and patients assessed, type of study (prospective or retrospective), and surgery (cataract or RLE), follow-up, patient ages, IOL power (spherical, and cylindrical for toric lenses), axial length, and the formula used to calculate the IOL power. Khoramnia et al. (16) had the largest sample of eyes out of all the 18 studies, with 106, while Ang (15) and Khoramnia et al. (16) reported the longest follow-up period (24 months). Most of the studies were prospective and involved cataract patients. It should be noted that three studies combined lenses in the same patient: Nagy et al. (7) implanted the FineVision POD F IOL in one eye and the FineVision HP IOL in the contralateral eye (randomized); Kim et al. (14) used the FineVision Triumf IOL (BVI Inc., Waltham, United States) in one eye and the FineVision HP IOL in the contralateral eye; and, finally, Danzinger et al. (18) put the Isopure IOL (BVI Inc., Waltham, United States) in the dominant eye and the FineVision HP IOL in the contralateral eye. In the discussion, for these studies, only monocular outcomes, related to the FineVision HP IOL, were taken into account. Considering all the studies, a total of 1,028 eyes from 632 patients were implanted with the FineVision HP IOL (192 eyes specifically implanted with the toric FineVision HP IOL). The average spherical IOL power across all the studies was 21.85 D, but the individual IOL values ranged from 16.37 D (21) to 23.50 D (23); for toric lenses this figure was 1.74 D, ranging from 1.43 D (24) to 1.94 D (19). Different IOL power calculation formulas were used but the Barrett Universal II was the most common. For toric lenses, the cylinder power and target axis were mainly calculated using the online FineVision Toric Calculator available at https://www.physioltoric.eu.

**Table 1:** Peer-reviewed clinical publications using the FineVision HP intraocular lens.

Authors (year) [country]	Eyes (patients)	Type (Surgery)	Follow-up (months)	Age (years)	Spherical IOL power (D)	Cylindrical IOL power (D)	Axial length (mm)	Formula for IOL power calculation
Nagy et al. (7) (2019) [Hungary]	25 (25)^a^	Prospective (Cataract)	6	58.8 ± 7.8 (43 to 78)	NR	–	NR	NR
Martínez de Carneros-Llorente et al. (8) (2019) [Spain]	80 (40)	Prospective (NR)	6	68.23 ± 5.57 (56 to 74)	24.14 ± 2.58 (17 to 23.50)	–	23.87 ± 0.70 (22.60 to 25.19)	SRK/T
Vinas et al. (9) (2020) [Spain]	20 (10)	Prospective (NR)	1	64.56 ± 3.52 (53 to 71)	23.15 ± 1.42 (21 to 26)	–	NR	NR
Poyales et al. (10) (2020) [Spain]	50 (25)	Prospective (Cataract)	1–3	66 ± 6.9 (52 to 83)	22.6 ± 2.0 (17 to 26)	–	23.28 ± 0.77 (21.81 to 25.08)	Barrett
Mayer et al. (11) (2022) [Germany]	2 (1)^b^	Prospective (Aphakia)	3	56	27	–	NR	NR
Garzón et al. (12) (2022) [Spain]	48 (48)	Prospective (Cataract)	1	67.7 ± 7.1 (NR)	NR	–	NR	NR
Benyoussef et al. (13) (2022) [France]	42 (21)	Prospective (Cataract+RLE)	1	57.81 ± 6.31 (44 to 70)	23.40 ± 3.56 (NR)	–	23.04 ± 1.08 (20.34 to 25.29)	Barrett Universal II
Kim et al. (14) (2022) [South Korea]	212 (106)^c^	Retrospective (Cataract)	6–10 weeks	57.5 ± 5.8 (42 to 70)	21.1 ± 2.00 (NR)^d^	–	23.64 ± 0.79 (NR)^d^	Barrett Universal II
Mori et al. (15) (2022) [Japan]	46 (23)	Prospective (Cataract)	6	71.3 ± 5.9 (56 to 82)	20.54 ± 3.68 (10 to 26)	–	23.66 ± 1.04 (22.15 to 26.68)	SRK/T and Barrett Universal II
Ang (16) (2023) [Philippines]	44 (22)	Prospective (Cataract)	24	67.9 ± 6.7 (58 to 79)	21.2 ± 1.9 (16 to 24.50)	–	23.62 ± 0.70 (22.04 to 24.75)	Barrett Universal II
Khoramnia et al. (17) (2023) [Germany]	112 (56)	Prospective (Cataract)	24	63.6 ± 9.3 (45 to 84)	22.40 ± 3.20 (11.50 to 29)	–	23.42 ± 1.11 (21.27 to 27.86)	Barrett True K
Danzinger et al. (18) (2023) [Austria]	50 (25)^e^	Prospective (Cataract)	6	66.44 ± 8.71 (53 to 81)	22.38 ± 4.20 (10 to 28.50)	–	23.30 ± 1.12 (21.63 to 27.20)	SRK/T, Haigis, Hoffer Q and Barrett Universal II
Daya and Espinosa Lagana (19) (2024) [United Kingdom]	62 (34) [64 toric IOLs]	Retrospective (Cataract+RLE)	6 weeks	62.39 ± 6.96 (51 to 80)	20.04 ± 5.74 (10 to 33.50)	1.94 ± 0.87 (1 to 4.50)	23.86 ± 1.75 (19.92 to 27.43)	Holladay 2, and the FineVision Toric Calculator
Akahoshi (20) (2024) [Japan]	45 (29)	Retrospective (Cataract)	3	68.52 ± 9.98 (33 to 80)	17.37 ± 3.78 (10.50 to 24)	–	24.71 ± 1.26 (23.01 to 27.39)	Barrett Universal II
Akahoshi (21) (2024) [Japan]	66 (39) [66 toric IOLs]	Retrospective (Cataract)	3	67.73 ± 10.74 (44 to 86)	16.37 ± 3.77 (10 to 24)	1.80 ± 0.99 (1 to 5.25)	25.06 ± 1.38 (22.38 to 27.63)	Barrett Universal II
Akahoshi (22) (2024) [Japan]	106 (53) [42 non-toric and 64 toric IOLs]	Prospective (Cataract)	3	66.66 ± 10.76 (33 to 86)	16.53 ± 3.78 (10 to 24)	1.79 ± 0.97 (1 to 5.25)	25.01 ± 1.35 (22.38 to 27.63)	Barrett Universal II
Nagy et al. (23) (2024) [Hungary]	51 (26)	Prospective	6	55.4 ± 7.0 (45 to 72)	23.5 ± 3.2 (16 to 29.50)	–	23.23 ± 1.11 (20.99 to 25.48)	Barrett Universal II
Bosc et al. (24) (2024) [France]	98 (49) [62 non-toric and 36 toric IOLs]	Retrospective (Cataract)	1–3	61.38 ± 6.42	21.85 ± 2.70 (14.50 to 28)	1.43 ± 0.44 (1 to 2.25)	23.49 ± 0.91 (21.44 to 26.14)	Barrett, and the FineVision Toric Calculator

Values reported as the mean ± standard deviation (range); D, diopters; IOL, intraocular lens; NR, not reported; RLE, refractive lens exchange.

a Mix-and-match: FineVision POD F / FineVision HP.

b 1 eye combined with Customflex.

c Mix-and-match: FineVision Triumf / FineVision HP.

d including eyes implanted with the Triumf IOL.

e Mix-and-match: Isopure / FineVision HP.

Table 2 describes in detail the refractive outcomes found in the different studies where data is available. Specifically, it shows the mean SE ± standard deviation value, the percentage of eyes with a SE of ±0.50 D and ± 1.00 D, the mean ± standard deviation value for the refractive cylinder, and the percentage of eyes with a refractive cylinder of ≤0.50 D and ≤ 1.00 D.

**Table 2:** Refractive outcomes obtained in peer-reviewed publications using the FineVision HP intraocular lens.

Authors	Mean SE (D)	SE ± 0.50 D (%)	SE ± 1.00 D (%)	Mean cylinder (D)	Cylinder ≤ 0.50 D (%)	Cylinder ≤ 1.00 D (%)
Nagy et al. (7)	0.05 ± 0.21	100	100	−0.18 ± 0.41	88	96
Martínez de Carneros-Llorente et al. (8)	−0.02 ± 0.46	–	–	–	–	–
Vinas et al. (9)	–	55	100	–	80	100
Poyales et al. (10)	0.23	90	92	–	–	–
Mayer et al. (11)^a^	–	–	–	–	–	–
Garzón et al. (12)	0.09 ± 0.42	–	–	−0.28 ± 0.34	–	–
Benyoussef et al. (13)	0.14 ± 0.64	73	92	–	–	–
Kim et al. (14)	−0.01 ± 0.30	–	–	−0.25 ± 0.27	–	–
Mori et al. (15)	−0.22 ± 0.38	74	98	–	82	100
Ang (16)	0.14	75	100	−0.54	–	–
Khoramnia et al. (17)	−0.02	81.8	100	−0.30	–	–
Danzinger et al. (18)	0.03 ± 0.43	–	–	–	–	–
Daya and Espinosa Lagana (19)	0.09 ± 0.39	88.71	100	−0.15 ± 0.24	93.54	100
Akahoshi (20)	−0.01 ± 0.22	97.78	100	−0.08 ± 0.24	91.11	100
Akahoshi (21)	0.00 ± 0.21	98.48	100	−0.08 ± 0.23	95.45	100
Akahoshi (22)	0.00 ± 0.22	98.11	100	−0.07 ± 0.23	93.40	100
Nagy et al. (23)	0.06 ± 0.42	72	94	−0.50 ± 0.33	–	–
Bosc et al. (24)	0.04 ± 0.37	–	–	−0.31 ± 0.26	–	–

Values reported as the mean ± standard deviation (when available); –: not reported; SE, spherical equivalent; D, diopters.

a Case report.

Table 3 provides data on, where available, the monocular UCVA and CDVA, DCIVA at 80, 70, 66 and 60 cm, and DCNVA at 40 and 35 cm. For some studies binocular visual acuity is indicated. Table 4 shows the percentage of eyes achieving a monocular visual acuity of ≥20/16, ≥20/20, ≥20/25, and ≥ 20/32 at distance, intermediate (80, 70, 66, and 60 cm) and near (40 and 35 cm). As in Table 3, some studies reported cumulative binocular visual acuity outcomes.

**Table 3:** Monocular visual acuity logarithm of the minimum angle of resolution (logMAR) outcomes at different distances obtained in peer-reviewed publications using the FineVision HP intraocular lens.

Authors	UDVA	CDVA	UIVA (80 cm)	DCIVA (80 cm)	UIVA (70 cm)	DCIVA (70 cm)	UIVA (66 cm)	DCIVA (66 cm)	UIVA (60 cm)	UNVA (40 cm)	DCNVA (40 cm)	UNVA (35 cm)	DCNVA (35 cm)
Nagy et al. (7)	0.00 ± 0.07	−0.04 ± 0.08	–	–	0.04 ± 0.09	0.04 ± 0.09	–	–	–	–	–	0.06 ± 0.08	0.04 ± 0.07
Martínez de Carneros-Llorente et al. (8)	0.05 ± 0.47	−0.02 ± 0.04	–	–	–	–	–	–	–	–	–	–	–
Vinas et al. (9)	0.06 ± 0.16	−0.03 ± 0.09	–	–	–	–	–	–	–	–	–	–	–
Poyales et al. (10)	0.01 ± 0.08	−0.03 ± 0.03	–	0.08 ± 0.10	–	–	–	–	–	–	0.13 ± 0.11	–	–
Mayer et al. (11)^a^	–	–	–	–	–	–	–	–	–	–	–	–	–
Garzón et al. (12)	0.08 ± 0.09	0.01 ± 0.03	–	–	–	–	–	–	–	–	–	–	–
Benyoussef et al. (13)	0.09 ± 0.14	−0.05 ± 0.07	–	–	0.04 ± 0.10	−0.03 ± 0.07	–	–	–	–	–	0.12 ± 0.10	−0.04 ± 0.09
Kim et al. (14)	0.03 ± 0.04	0.01 ± 0.02	–	–	–	–	–	–	–	0.04 ± 0.06	–	–	–
Mori et al. (15)	−0.03 ± 0.08	−0.11 ± 0.02	0.07 ± 0.11	0.02 ± 0.08	–	–	–	–	–	0.08 ± 0.09	0.06 ± 0.09	–	–
Ang (16)^b^	–	−0.01 ± 0.05	–	–	–	0.04 ± 0.08	–	–	–	–	–	–	0.06 ± 0.09
Khoramnia et al. (17)^b^	–	−0.07 ± 0.08	–	–	–	0.00 ± 0.10	–	–	–	–	–	–	0.04 ± 0.10
Danzinger et al. (18)	–	−0.03 ± 0.09	–	0.18 ± 0.11	–	–	–	–	–	–	0.20 ± 0.15	–	–
Daya and Espinosa Lagana (19)	0.01 ± 0.06	−0.01 ± 0.04	0.00 ± 0.07	–	–	–	–	–	0.03 ± 0.07	0.04 ± 0.07	–	–	–
Akahoshi (20)	−0.05 ± 0.07	−0.07 ± 0.06	0.18 ± 0.14	0.16 ± 0.14	–	–	0.20 ± 0.15	0.19 ± 0.15	–	0.04 ± 0.10	0.03 ± 0.10	–	–
Akahoshi (21)	−0.06 ± 0.07	−0.07 ± 0.06	0.19 ± 0.12	0.19 ± 0.12	–	–	0.18 ± 0.12	0.18 ± 0.12	–	0.03 ± 0.10	0.02 ± 0.08	–	–
Akahoshi (22)	−0.05 ± 0.07	−0.07 ± 0.06	–	–	–	–	–	–	–	–	–	–	–
Nagy et al. (23)	0.01 ± 0.09	−0.06 ± 0.08	–	–	0.12 ± 0.10	0.10 ± 0.09	–	–	–	–	–	0.15 ± 0.10	0.12 ± 0.13
Bosc et al. (24)	0.03 ± 0.09	–	–	–	–	–	0.29 ± 0.11	–	–	0.19 ± 0.16	–	–	–

Values reported as the mean ± standard deviation; –, not reported; UDVA, uncorrected distance visual acuity; CDVA, corrected distance visual acuity; UIVA, uncorrected distance intermediate visual acuity; DCIVA, distance-corrected intermediate visual acuity; UNVA, uncorrected near visual acuity; DCNVA, distance-corrected near visual acuity.

a Case report.

b Binocular visual acuity.

**Table 4:** Percentage of cumulative monocular visual acuity [≥20/16 (−0.1 logMAR), ≥20/20 (0 logMAR), ≥20/25 (0.1 logMAR), and ≥ 20/32 (0.2 logMAR)] outcomes at different distances obtained in peer-reviewed publications using the FineVision HP intraocular lens.

Authors	UDVA	CDVA	UIVA (80 cm)	DCIVA (80 cm)	UIVA (70 cm)	DCIVA (70 cm)	UIVA (66 cm)	DCIVA (66 cm)	UIVA (60 cm)	UNVA (40 cm)	DCNVA (40 cm)	UNVA (35 cm)	DCNVA (35 cm)
Nagy et al. (7)	16, 76, 100, 100	32, 96, 100, 100	–	–	–	12, 64, 88, 100	–	–	–	–	–	–	4, 60, 92, 100
Martínez de Carneros-Llorente et al. (8)	–	–	–	–	–	–	–	–	–	–	–	–	–
Vinas et al. (9)	–	–	–	–	–	–	–	–	–	–	–	–	–
Poyales et al. (10)	0, 62.5, 95.8, 95.8	0, 87.5, 100, 100	–	8.3, 29.2, 79.2, 100	–	4, 40, 88, 100	–	–	–	–	0, 16.7, 70.8, 87.5	–	4, 48, 88, 96
Mayer et al. (11)^b^	–	–	–	–	–	–	–	–	–	–	–	–	–
Garzón et al. (12)	–	–	–	–	–	–	–	–	–	–	–	–	–
Benyoussef et al. (13)	7, 29, 45, 88	38, 86, 98, 100	–	–	–	–	–	–	–	–	–	–	–
	^a^29, 52, 81, 100	^a^62, 90, 98, 100											
Kim et al. (14)	–	–	–	–	–	–	–	–	–	–	–	–	–
Mori et al. (15)	35, 96, 100, 100	74, 100, 100, 100	9, 74, 96, 100	4, 74, 100, 100	–	–	–	–	–	9, 52, 96, 96	9, 74, 96, 100	–	–
Ang (16)^a^	16.7, 66.7, 100, 100	16.7, 91.7, 100, 100	–	–	–	8.3, 58.3, 91.7, 100	–	–	–	–	–	–	8.3, 50, 83.3, 100
Khoramnia et al. (17)	13.6, 36.4, 69.7, 90.9	27.3, 72.7 97, 100	–	–	3, 33.3, 62.1, 86.4	0, 16.7, 59.1, 87.9	–	–	–	–	–	0, 21.2, 42.4, 74.2	4.7, 25, 57.8, 84.4
	^a^36.4, 72.7, 93.9, 100	^a^48.5, 90.9, 100, 100			^a^30.3, 69.7, 90.9, 100	^a^27.3, 60.6, 93.9, 97						^a^15.2, 45.5, 69.7, 93.9	^a^18.8, 46.9, 97.5, 96.9
Danzinger et al. (18)	–	–	–	–	–	–	–	–		–	–	–	–
Daya and Espinosa Lagana (19)	–, 82.26, 98.39, 100	–, 95.16, 100, 100	–, 80,65, 100, 100	–	–	–	–	–	–, 67.4, 95.16, 100	–, 67.4, 90.32, 100	–	–	–
Akahoshi (20)	66.6, 86.6, 100, 100	73.3, 95.5, 100, 100	0, 15.5, 55.5, 68.8	2.2, 20, 60, 71.1	–	–	4.4, 15.5, 37.7, 68.8	4.4, 15.5, 40, 71.1	–	17.7, 53.3 86.6, 95.5	20, 53.3, 86.6, 97.7	–	–
Akahoshi (21)	68.1, 87.8, 100, 100	75.7, 90.9, 100, 100	0, 9, 40.9, 72.7	1.5, 9, 42.2, 74.2	–	–	1.5, 13.6, 33.4, 77.2	1.5, 13.6, 34.8, 78.7	–	19.7, 59 87.8, 98.4	19.7, 60.6, 89.3, 100	–	–
Akahoshi (22)	–	–	–	–	–	–	–	–	–	–	–	–	–
Nagy et al. (23)	–	62, 92, 98, 98	–	–	–	4, 28, 80, 96	–	–	–	–	–	–	2, 42, 62, 84
Bosch et al. (24)	–	–	–	–	–	–	–	–	–	–	–	–	–

UDVA, uncorrected distance visual acuity; CDVA, corrected distance visual acuity; UIVA, uncorrected distance intermediate visual acuity; DCIVA, distance-corrected intermediate visual acuity; UNVA, uncorrected near visual acuity; DCNVA, distance-corrected near visual acuity; –, not reported.

a Binocular visual acuities.

b Case report.

Monocular or binocular defocus curves were measured in some studies (7, 8, 11, 13–18, 23) and the outcomes obtained were represented graphically in curves where visual acuity changed with vergence (defocus). In order to make a general comparison between the studies we created Table 5. This shows the diopter range in which patients, under monocular or binocular conditions, showed a visual acuity of ≥20/32 (0.2 logMAR). It should be noted that these values were estimated from the curves published by the different authors. The ranges varied from 3.00 to 4.00 D under monocular conditions (7, 8, 14, 18) to 4.25 to 5.00 D under binocular conditions (13, 15–17, 23, 24).

**Table 5:** Defocus curve outcomes obtained in peer-reviewed publications using the FineVision HP intraocular lens.

Authors	Diopter range with a visual acuity ≥ 20/32 (0.2 logMAR)^a^	
	Monocular	Binocular
Nagy et al. (7)	3.00 (between +0.50 and −3.00)	–
Martínez de Carneros-Llorente et al. (8)	4.00 (between +0.75 and −3.25)	
Benyoussef et al. (13)	–	4.50 (between +1.00 and −3.50)
Kim et al. (14)^b^	3.75 (between +0.75 and −3.00)	4.25 (between +1.00 and −3.25)
Mori et al. (15)	–	4.75 (between +1.00 and −3.75)
Ang (16)	–	4.50 (between +1.00 and −3.50)
Khoramnia et al. (17)	–	4.50 (between +1.00 and −3.50)
Danzinger et al. (18)	4.00 (between +0.75 and −3.25)	–
Nagy et al. (23)	–	4.75 (between +1.00 and −3.75)
Bosc et al. (24)	–	5.00 (between +1.25 and −3.75)

–, not reported.

a Values estimated from the curves published.

b For binocular conditions authors performed a mix-and-match technique: FineVision Triumf / FineVision HP.

**Figure 2:**
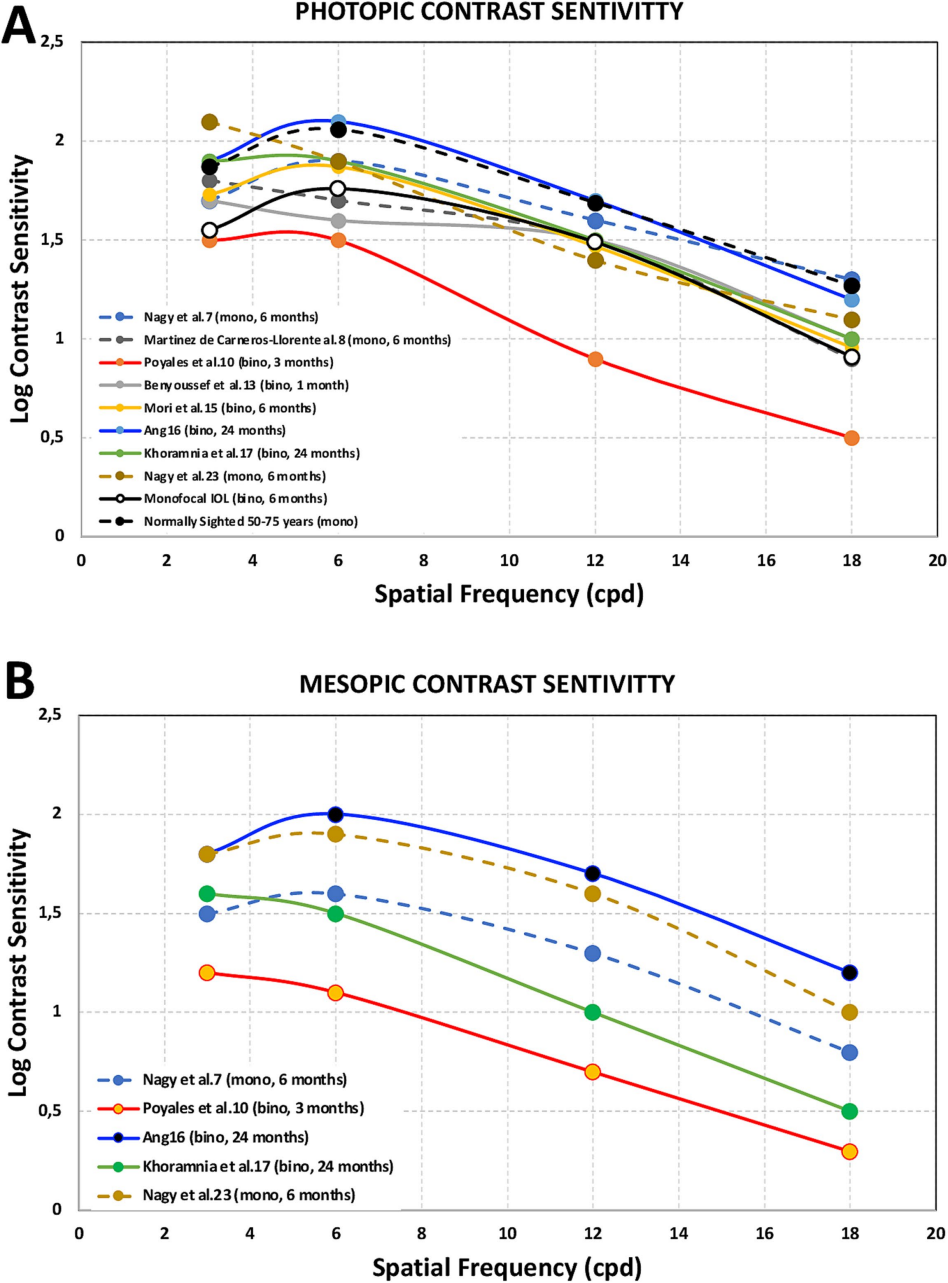
Photopic [85 cd/m^2^, **(A)**] and mesopic [3.5 (7, 10, 16, 17, 23) or 3 (8) cd/m^2^, **(B)**] contrast sensitivity outcomes obtained after FineVision HP intraocular lens implantation for spatial frequencies of 3, 6, 12 and 18 cycles per degree at different times post-surgery (from 1 to 24 months) from Nagy et al. (7), Martínez de Carneros-Llorente et al. (8), Poyales et al. (10), Benyoussef et al. (13), Mori et al. (15), Ang (16), Khoramnia et al. (17), and Nagy et al. (23). Note that these values were estimated from the graphs published in the different studies. Mean binocular values from a monofocal intraocular lens group (33) and monocular values for normal patients in the 50–75 years range (34) were also included for comparison.

The optical quality of eyes implanted with this IOL model was measured by different authors. Vinas et al. (9) measured the longitudinal chromatic aberration (LCA) using psychophysical methods, Poyales et al. (10) employed the modulation transfer function (MTF) and the Strehl ratio using the OQAS instrument (Visiometrics SL, Terrassa, Spain), Danzinger et al. (18) applied internal higher order aberrations (HOAs) using the Sirirus topographer and the Peramis aberrometer (SCHWIND eye-tech-solutions, Kleinostheim, Germany). Contrast sensitivity was measured in several studies, both under photopic (85 cd/m^2^) and mesopic (3 or 3.5 cd/m^2^) conditions at different times post-surgery (from 1 to 24 months) (7, 8, 10, 13, 15–17, 23). Figure 2 shows the estimated values reported by these studies obtained from the graphs published under photopic (Figure 2A) and mesopic (Figure 2B) conditions.

**Figure 3:**
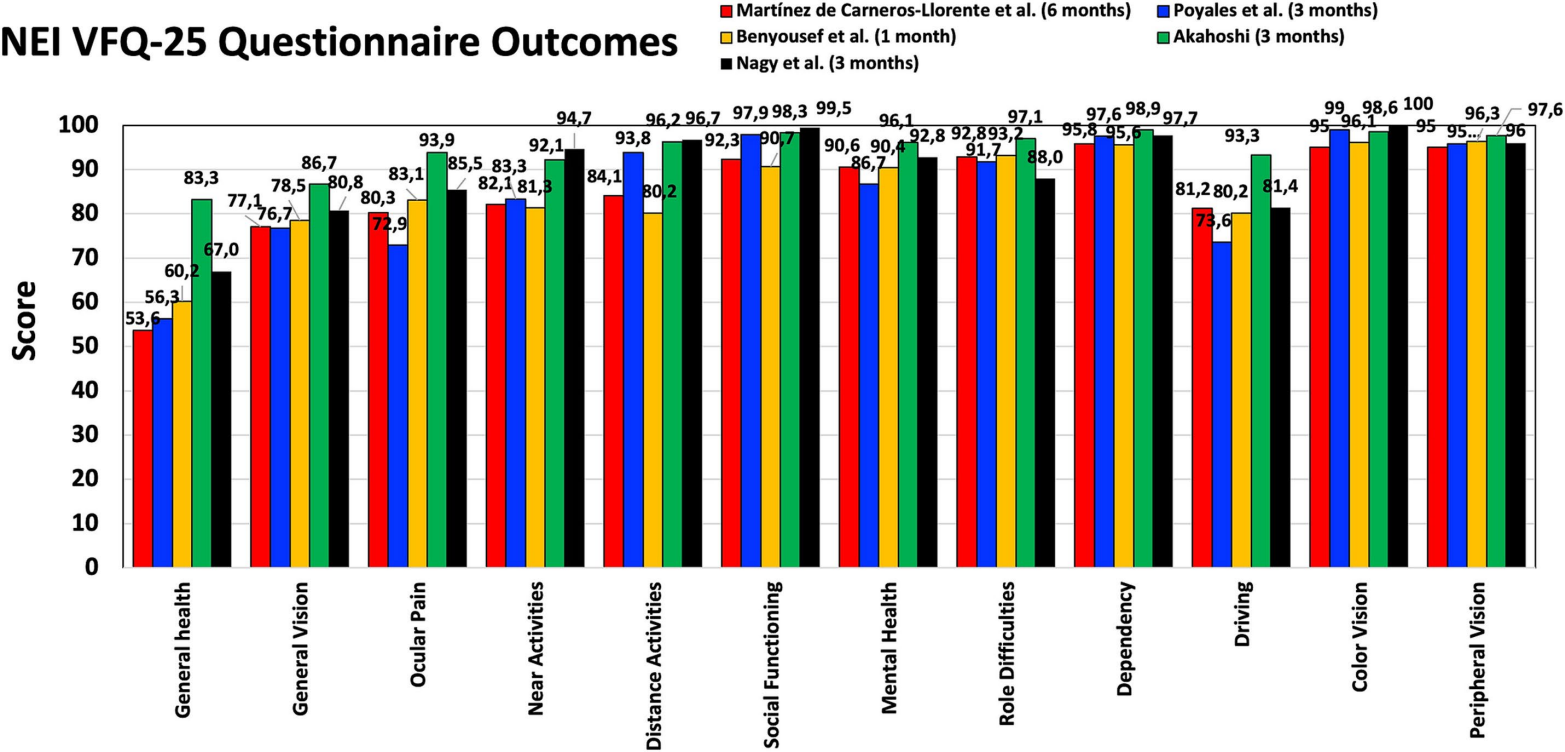
Patient-reported outcomes after FineVision HP intraocular lens implantation: National Eye Institute Visual Function Questionnaire-25 (NEI VFQ-25) scores obtained by Martínez de Carneros-Llorente et al. (8) at 6 months, Poyales et al. (10) at 3 months, Benyoussef et al. (13) at 1 month, and Akahoshi (22) and Nagy et al. (23) at 3 months.

**Figure 4:**
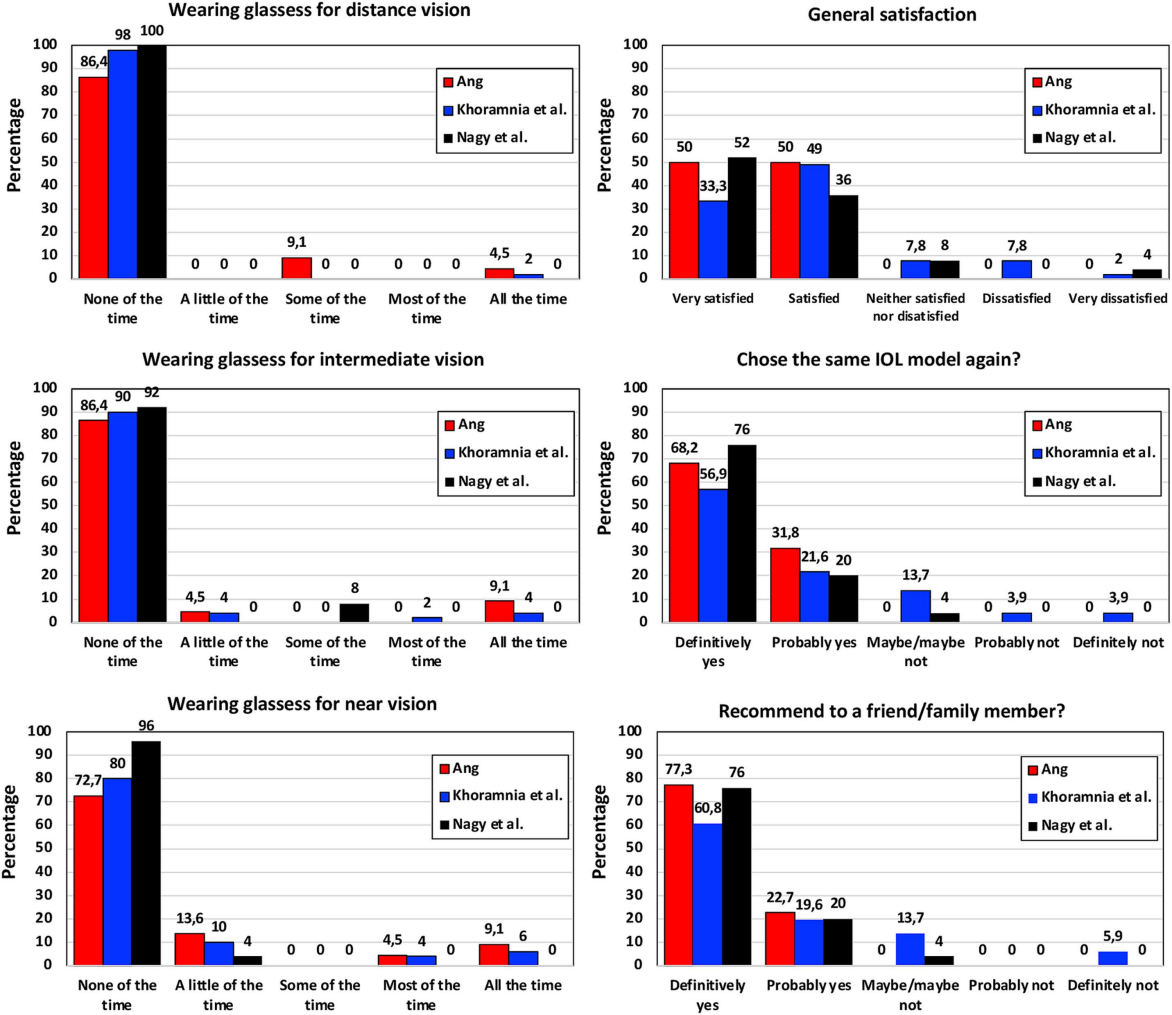
Patient-reported outcomes after FineVision HP intraocular lens implantation: self-assessment questionnaire percentages obtained by Ang (16), Khoramnia et al. (17) and Nagy et al. (23) at 3-months post-surgery.

PROQ were analyzed in several studies using different questionnaires. Figure 3 shows the mean scores obtained by Martínez de Carneros-Llorente et al. (8) at 6 months, Poyales et al. (10) at 3 months, Benyoussef et al. (13) at 1 month, and Akahoshi (22) and Nagy et al. (23) both at 3 months, using the National Eye Institute Visual Function Questionnaire-25 (NEI VFQ-25). Akahoshi (22) also administered the Catquest-9SF questionnaire and the Patient Reported Spectacle Independence Questionnaire (PRSIQ) to their cohort. Figure 4 shows the percentages reported in a self-assessment questionnaire used by Ang (16), Khoramnia et al. (17) and Nagy et al. (23) at 3 months post-surgery, asking about the patients’ need to wear glasses for various distances, their general level of satisfaction, whether they would choose the same IOL model again, and if they would recommend this IOL to a friend or family member. Kim et al. (14) and Danzinger et al. (18) also reported outcomes using a self-assessment questionnaire and the VF-7 questionnaire but these two studies were not considered since the patients were implanted following a mix-and-match procedure (FineVision Triumf/FineVision HP IOLs (14), and Isopure/FineVision HP IOLs (18)). In relation to photic phenomena, Poyales et al. (10) studied halometry with the Halo v1.0 (University of Granada, Granada, Spain) at 3 months, and evaluated negative dysphotopsia by asking patients whether they perceived a dark shadow in their peripheral visual field at 1 month. At the same follow-up period, Benyoussef et al. (13) analyzed halometry using the halometry test (Aston University, Birmingham, United Kingdom) and asked patients about the frequency of halos and glare. Khoramnia et al. (17), at a longer follow-up period (6 months), used a Halo and Glare Simulator (Eyeland Design Network GmbH, Vreden, Germany) to assess this subjective perception. The same test was used by Danzinger et al. (18) but the results were not considered in this review due to the mix-and-match procedure employed.

Finally, we found that only 2 of the different studies evaluated reported adverse effects. Poyales et al. (10) indicated that dry eye was diagnosed in 2 eyes, and 1 eye developed posterior vitreous detachment 3 months after surgery. Those authors also indicated that no glistening was observed and that 1 patient showed posterior capsule opacification classified as “mild” but no Nd:YAG treatment was needed. Additionally, Daya and Espinosa Lagana (19) found that 1 eye of 1 patient developed capsular phimosis with a mild hyperopic shift from posterior lens displacement, resolved by a Nd:YAG anterior capsulotomy, which resulted in reduced hyperopia. Nagy et al. (23) reported 3 adverse events and 4 serious adverse events. All of them were classified as not related to the medical device, and 2 were classified as related to the procedure. These 2 were 1 macular edema and 1 corneal edema, both resolved without sequelae.

## 4 Discussion

Correction of presbyopia during cataract surgery with various intraocular lens options is becoming more popular and prevalent among patients attending our centers. The increasing demand for this type of surgery pushes us to look for presbyopia-correcting lenses that can provide our patients a wide range of excellent visual acuity and reduce their spectacle dependence. As we have highlighted, diffractive trifocal IOLs are one of the best solutions for this. Specifically, in those who do not have underlying pathology that would prevent excellent results. Our review analyzes the outcomes of one of these lenses, the hydrophobic trifocal diffractive FineVision HP IOL. Specifically, this section now looks at the impact of the outcomes obtained after implantation of this IOL.

### 4.1 Refractive error

Residual refractive error can lead to dissatisfaction following implantation of presbyopia correcting IOLs. Published studies analyzed the FineVision HP IOL, and the accuracy of hitting refractive targets. Table 2 shows the detailed values obtained. We can see that the mean postoperative SE values reported were close to emmetropia and never greater than a quarter of a diopter. Almost all the eyes from the different studies were within 1.00 D of SE. These studies involved follow-up periods from 1 month to 2 years, demonstrating the stability of the procedure. For the longer follow-ups the percentages for ±0.50 D were 75% (16) and 81.8% (17), being 100% for ±1.00 D (16, 17). The mean postoperative refractive cylinder values were also excellent with the main values being less than a quarter of a diopter and only two studies reporting about half a diopter (16, 23). Only Daya and Espinosa Lagana (19) (64 eyes at 6 weeks), Akahoshi (21, 22) (66 and 64 eyes at 3 months) and Bosc et al. (24) (36 eyes at 1–3 months) included the toric model in their cohorts, from 1.00 up to 5.25 D of cylinder. Specifically, in these studies, the accuracy for astigmatism correction was excellent: a cylinder value of ≤0.50 D for 93.40 to 95.45% of eyes, and ≤ 1.00 D in 100%. A high percentage of authors used the Barrett Universal II formula for the IOL power calculation (see Table 1 for a detailed description). A recent systematic review for IOL power calculation formulas concluded that, of all vergence formulas, this is currently the most often reported and most precise (25). A comparison of 12 formulas for the hydrophilic FineVision Micro F IOL in 172 eyes revealed that the Barrett Universal II formula provides excellent overall accuracy for eyes (86.55% ±0.50 D and a mean absolute error of 0.285 D), it being the most accurate for medium axial length eyes (26). The cohorts analyzed in our review mainly involved mean axial lengths about 23 mm, except for Japanese eyes which had longer values, around 25 mm (see Table 1). A questionnaire-based study conducted among members of the Japanese Society of Cataract and Refractive Surgery over the past 20 years revealed that the use of trifocal IOLs has particularly increased, and, in terms of IOL power calculations, the Barrett Universal II formula has been gaining popularity when there is no post-laser vision correction (27). We should therefore consider that the use of this formula with the FineVision HP IOL provides excellent refractive outcomes. However, it should be noted that some authors have used other formulas, such as the SRK/T, Haigis, Hoffer Q, or Holladay 2, for example, also reporting good refractive accuracy. For this reason, we believe that surgeons should consider applying these popular formulas to calculate the IOL power. To make a comparison with other trifocal IOLs, it is interesting to discuss the outcomes published recently in a systematic review and meta-analysis of 22 studies reporting data for trifocal and EDOF IOLs (2). This study considered publications reporting on the FineVision, the AcrySof IQ PanOptix (Alcon Labs, Fort Worth, USA), and the AT LISA tri (Carl Zeiss Meditec, Jena, Germany) IOLs; here the mean SE postoperative outcomes reported varied from −0.39 to 0.25 D, values which are in agreement with the mean SE values found in the FineVision HP IOL studies (Table 2).

Rotational stability was only reported by Daya and Espinosa Lagana (19), who showed a mean IOL rotation of 5.84 ± 6.41° from the intented axis of implantation, noted on the operative form, to the orientation of the lens on internal aberrometry (implied rotation) at the last postoperative visit (6 weeks). 38 eyes (61.3%) were within 5° of the intended orientation, 18 (29%) were between 6 and 10°, and 6 (9.7%) were more than 10° away from the intended axis. As these authors discussed, this method is not as accurate as directly observing the IOL’s position with a dilated pupil, as it is highly dependent on the quality of the test performed and prone to error from IOL tilt. Based on the good refractive cylindrical outcomes in that cohort, the authors suggested that in reality there was minimal, if any, cylindrical correction efficacy reduction in spite of the slightly higher rotation value found. The method used may be the source of this rotation value. Other studies, using the same platform with the hydrophilic version of the lens (FineVision POD FT IOL) reported better rotational stability outcomes. For example, Vandekerckhove (28), using images captured from a camera for 37 eyes, indicated mean values of 2.56 ± 2.22° and 2.55 ± 2.62° at 6 and 12 months postoperatively, respectively; and Ang (29), using a slit lamp on 187 eyes, found mean values of 1.77 ± 1.82°, 1.83 ± 1.85°, and 2.00 ± 2.42°, at 4 to 6 months, 11 to 13 months, and 21 to 26 months postoperatively, respectively. Furthermore, Ruiz-Mesa et al. (30), using this model in eyes with high levels of corneal astigmatism (cylindrical IOL power of 3.75 D or greater), with the longest follow-up period (5 years), found no significant rotation in their cohort. It is therefore expected that the toric version of the FineVision HP IOL will demonstrate good rotational stability.

### 4.2 Visual acuity

As mentioned in the results section, visual acuity was evaluated in all the studies analyzed. It should be considered that for trifocal lenses, in addition to distance visual acuity, intermediate and near visual acuities should be measured in order to determine how these lenses provide good visual acuity at different distances. All the studies detailed in Table 3 reported CDVA values, in almost all cases these being better than 20/20. Also, almost all the studies provided UDVA outcomes, which were also good. It should be noted that the Bayesian network meta-analysis comparing the outcomes of different presbyopia-correcting IOLs frequently used in clinical practice found that, for UDVA, all multifocal IOLs were comparable with monofocal IOLs (1). The systematic review and meta-analysis by Karam et al. (2) of several trifocal IOLs showed mean UDVA values ranging from −0.12 to 0.11 logMAR, and mean CDVA values ranging from −0.2 to 0.06 logMAR, these being similar to those reported in this review (see Table 3). The outcomes for the FineVision HP IOL at far distance were stable both in the short-term (1 month) (9, 12, 13, 24) and long-term (24 months) (16, 17). For other distances, the visual acuity can be measured at different optotype locations. Table 3 shows that monocular UIVA and DCIVA was measured at 80, 70, 66 and 60 cm in different clinical studies; it was usually measured at 80 and 70 cm, with DCIVA values ranging from 0.02 (15) to 0.19 (21) logMAR at 80 cm (about 20/20 to 20/32), and from −0.03 (13) to 0.12 (23) logMAR at 70 cm (about 20/20 to 20/25). None of the studies analyzed the intermediate visual acuity at different distances; however, if we make a comparison, it seems that better outcomes were found for 70 cm compared to 80 cm. Monocular UIVA measured at 60 cm was also close to 20/20 (0.03 logMAR) (19). This tendency is related to the intermediate focus created by the IOL (+1.75 D). For near vision, a monocular DCNVA at both 40 cm and 35 cm was good, ranging from 0.02 (21) to 0.20 (18) logMAR (about 20/20 to 20/32), and from 0.12 (23) to −0.04 logMAR (13), respectively (about 20/20 to 20/25). Again, the better outcomes for closer distances are related to the near focus of the IOL (+3.50 D). It should be noted that, in general, distance visual acuity was good, better than or equal to 20/20, and both intermediate and near vision were quite similar, maintaining a high level, ranging from 20/20 to 20/32. Karam et al. (2) reported similar outcomes, with mean UIVA values ranging from −0.01 to 0.29 logMAR, and a mean DCIVA ranging from 0.006 to 0.24 logMAR. For near vision, those authors found a mean UNVA ranging from −0.03 to 0.18 logMAR, and a mean DCNVA ranging from 0.06 to 0.19 logMAR.

Mean visual acuity values correlate with cumulative visual acuity outcomes. Table 4 shows the percentages for monocular and, in some studies, binocular visual acuity at different distances. Note that the percentages for ≥20/20 (0 logMAR) are, in most cases, between about 90 and 100% for CDVA. As expected, slightly lower values than for CDVA are found for DCIVA at 80, 70 and 66 cm, and for DCNVA at both 40 and 35 cm. However, these values show that this lens offers good outcomes for intermediate and near vision. For example, Ang (16) and Khoramnia et al. (17), with the longest follow-up periods (2 years) showed similar binocular cumulative visual acuity values: about 60, 90, and 100% for ≥20/20 (0 logMAR), ≥20/25 (0.1 logMAR), and ≥ 20/32 (0.2 logMAR), respectively, at 70 cm, this being similar at 35 cm. Therefore, in terms of visual acuity performance this lens provides excellent vision at far distances, and good vision for intermediate and near distances.

### 4.3 Defocus curve

In relation to the defocus curve, which is an excellent metric for determining the visual performance of our patients at different vergences (distances), we have created Table 5 to show the diopter range in which patients show a visual acuity of ≥20/32 (0.2 logMAR). Note that these values were estimated from the defocus curves depicted by authors in their respective publications. This range give us a detailed value of the useful range of vision provided by this lens. In general, the defocus curves for this lens show a peak of visual acuity for distance vision (0 D of defocus, vergence), followed by a smooth reduction as negative vergence increases in value (negative defocus values) up to another peak located in near vision (about −2.5 D of defocus). The ranges obtained in Table 5, which can be defined as the depth-of-focus of the lens, reveal that this is between 3.00 to 4.00 D under monocular conditions (7, 8, 14, 18) to 4.25 to 5.00 D under binocular conditions (13, 15–17, 23, 24). This depth-of-focus increases about 1 D more for binocular conditions, which is the reality in day-to-day activities for our patients. Kim et al. (14) used a mix-and-match technique combining the FineVision HP IOL with the FineVision Triumf IOL obtaining a depth-of-focus under binocular conditions of 4.25 D, which is slightly lower than the value obtained under binocular conditions when implanted with bilateral FineVision HP IOLs (4.50/4.75 D). We therefore consider that bilateral implantation of the FineVision HP IOL is the best approach, providing a continuum of visual acuity at far, intermediate and near distances.

### 4.4 Optical quality

Vinas et al. (9) measured the LCA of the FineVision HP IOL using psychophysical methods. These authors aimed to provide insights into how the material of the lens and its multifocal design influenced the LCA of 20 implanted eyes. They found that the LCA was significantly higher for far vision than for intermediate and near vision (*p* < 0.05), and slightly higher for the hydrophobic lens than for the hydrophilic counterpart at far distances. Poyales et al. (10) recorded some optical metrics, such as the MTF, Strehl Ratio and HOAs, at 3 months post-surgery; when comparing the hydrophilic and hydrophobic FineVision IOLs they found that the MTF (25.14 versus 26.48) and Strehl Ratio (0.15 versus 0.16) were close to that of a normal population, and the tilt (0.24 versus 0.33 μm), HOAs (0.27 versus 0.40 μm) and spherical aberration (0.28 versus 0.26 μm) were similar for the two IOLs. In another study, Danzinger et al. (18) measured internal HOAs and compared the FineVision HP IOL with the Isopure IOL. These authors found that at a 5 mm pupil eyes implanted with the FineVision HP had significantly lower HOAs values (FineVision HP: 0.33 ± 0.10 μm; Isopure: 0.57 ± 0.11 μm; *p* < 0.01) with significantly increased negative spherical aberration in the Isopure eyes (Isopure: −0.40 ± 0.09 μm; FineVision HP: −0.05 ± 0.08 μm; *p* < 0.01). With a smaller pupil, 3 mm, the outcomes showed comparable HOAs in the two groups (Isopure: 0.18 ± 0.10 μm; FineVision HP: 0.14 ± 0.07 μm; *p* = 0.107) with a small but significantly greater negative spherical aberration in the Isopure eyes (Isopure: −0.04 ± 0.04 μm; FineVision HP: −0.02 ± 0.02 μm; p < 0.01). The increased spherical aberration value in the Isopure eyes correlates with the design of this surface which has been shown to differ from an aspheric monofocal lens (31). Note that Shack-Hartmann technology presents some problems testing diffractive IOLs (32) and the results might not be compared with that of non-diffractive IOLs.

### 4.5 Contrast sensitivity

It is considered that contrast sensitivity function assessment is the best parameter for measuring spatial vision limits, providing information about visual performance for a range of object scales. Its measurement under different lighting conditions, mainly photopic and mesopic, can inform us about the vision of our patients in these circumstances. Superimposed images due to multifocality (in-focus and out-of-focus images) may result in a reduced-contrast retinal image and, therefore, lower contrast sensitivity (33). Several studies have recorded the contrast sensitivity at different spatial frequencies after implantation of FineVision HP IOLs. Figure 2 shows the estimated curves obtained from the graphs published under photopic (top: 2A, 85 cd/m^2^) and mesopic (bottom: 2B, 3.5 or 3 cd/m^2^) conditions in these studies (7, 8, 10, 13, 15–17, 23). This figure also illustrates mean binocular contrast sensitivity in patients implanted with monofocal IOLs (33) and monocular mean values for normal patients in the 50–75 year age range (mean 63.9 years) (34) for comparison. As can be seen, the reported outcomes were good, especially in those studies where binocular measurements were taken and there were longer-follow-up periods compared to the monofocal IOLs and the normally sighted groups. Cao et al. (35), in their meta-analysis of 21 randomized controlled trials involving 2,951 subjects, observed lower contrast sensitivity in multifocal IOL patients; however, they indicated that the gap between these patients and those implanted with monofocal lenses was only 0.06 units, meaning the disadvantage of multifocal IOLs in terms of contrast sensitivity is not very great. The outcomes found here agree with that statement. It is interesting to note that in studies with longer follow-up periods better outcomes were found for all spatial frequencies and both illumination conditions (from 6 to 24 months). Indeed, it has been reported that the typical neuroadaptation process after multifocal IOL implantation is at least 3 months in terms of significantly reducing photic phenomena (36), and the maximum improvement is likely to be reached 12 months after surgery (37). We may observe a reduction in contrast sensitivity under mesopic conditions, particularly at higher spatial frequencies. This behavior agrees with classic data on the effect of luminance level on contrast sensitivity (38). In addition, it has been published that contrast sensitivity function might be significantly affected due to the light division that occurs in multifocal IOLs, especially in low-mesopic environments (36).

### 4.6 Patient-reported outcome questionnaires

Martínez de Carneros-Llorente et al. (8), Poyales et al. (10), Benyoussef et al. (13), Akahoshi (22) and Nagy et al. (23) used the NEI VFQ-25 questionnaire with their cohorts, reporting high scores for the different questions analyzed, mainly distance and near activities (Figure 3). The maximum score value was 100 and all the questions related to vision were around 80 or above in all the studies. Other studies involving the hydrophilic version of this lens reported similar outcomes: the median sub-score values were ≥ 80 for general, near and far vision, and driving 3 months post-surgery (39); mean values of 93.64 ± 4.16, 91.00 ± 13.78, 89.44 ± 13.54, 83.88 ± 14.95, and 89.76 ± 20.14 were reported for overall satisfaction, general vision, far activities, near activities, and driving, respectively, 6 months post-surgery (40). Poyales et al. (10) also evaluated the hydrophilic lens and found comparable outcomes (*p* > 0.05): 84.2, 89.9, and 93.8 mean values for general vision, near and distance activities, respectively. Martínez de Carneros-Llorente et al. (8) in their study also compared the outcomes of the FineVision HP IOL with two other trifocal IOLs, the AT LISA tri 839MP and the AcrySof IQ PanOptix. They found no statistically significant differences for any of the items on the questionnaire (*p* > 0.07), and considered that, independent of the IOL implanted, patient satisfaction was high. As indicated in the results section, Akahoshi (22) administered two other questionnaires to their sample. Using the Catquest-9SF, he found that 90.57% of patients reported no sight difficulties in their everyday-life, and all of them were “very or quite satisfied” with their sight. According to the PRSIQ outcomes, 98.11 and 98.11% of patients did not need glasses or contacts for far, intermediate and near vision, respectively. He concluded that patients bilaterally implanted with this IOL had high vision and health-related quality-of-life scores, with a high spectacle-independence rate and high patient satisfaction. The self-assessment questionnaire used by Ang (16), Khoramnia et al. (17) and Nagy et al. (23) 3 months post-surgery revealed that the use of glasses for distance, intermediate and near vision was very low (Figure 4). Indeed, for near vision a high percentage of the patients in the three studies reported using glasses “none of the time.” The general satisfaction level indicated that all the patients in the Ang (16) cohort, 82.3% in the Khoramnia et al. (17) sample and 88% in the Nagy et al. (23) were “very satisfied” and/or “satisfied.” Similarly, 100, 78.5 and 96% of patients answered “definitely yes” and/or “probably yes,” respectively, when asked whether they would choose the same IOL model again. Also, 100, 80.4 and 96%, respectively, would recommend this IOL to a friend or family member. In general, in line with the different PROQ outcomes published, we consider that this lens offers generally satisfying results in terms of performing everyday tasks.

### 4.7 Photic phenomena

The possible increase in photic phenomena after multifocal IOL implantation is a factor that should be kept in mind. Poyales et al. (10) studied halometry 3 months post-surgery and their results showed that the lens appeared not to introduce any additional problems to those reported for multifocal diffractive designs. They stated that 3 patients reported negative dysphotopsia on day 1 post-surgery but did not report this after 1 month. Khoramnia et al. (16), at a longer follow-up period (6 months), measured the size and intensity of the photic phenomena on a scale from 0 to 100, with halo size, halo intensity, glare size, and glare intensity being 46, 50.3, 13.1 and 22.8, respectively. They compared the IOL with its hydrophilic counterpart and found no statistically significant differences (*p* > 0.2). Low glare and halo values did not hinder the majority of the activities that patients carry out daily (18). However, we consider that further studies should be carried out to fully analyze photic phenomena.

### 4.8 Adverse events

Only 3 studies reported adverse events in their sample, these being dry eye in two eyes, a posterior vitreous detachment in one eye, a capsular phimosis in another eye, one macular edema and one corneal edema. The presence of glistening was not indicated by any of the different authors in the publications analyzed.

## 5 Conclusion

This assessment evaluated the outcomes of the FineVision HP IOL after its implantation. The results suggest that this IOL model provides accurate refractive outcomes and good visual acuity at distance, intermediate and near. Other performance metrics evaluated, such as optical quality measurement, contrast sensitivity analysis, and PROQ, also support the use of this lens. Additionally, of the clinical studies examined, only two reported adverse events, these being resolved during the follow-up. Future research with this lens should be carried out with larger samples and it should be compared with other hydrophobic trifocal diffractive lenses available on the market.
